# The Role of Green Infrastructure in Enhancing Flood Resilience: A Spatial Perspective

**DOI:** 10.1007/s00267-026-02470-9

**Published:** 2026-04-21

**Authors:** Jinhee Han, Yoomi Kim, Katsuya Tanaka

**Affiliations:** 1 Department of Public Administration, Ewha Womans University, Seoul, Republic of Korea; 2 Center of SEBIS (Strategic Solutions for Environmental Blindspots in the Interest of Society), Ewha Womans University, Seoul, Republic of Korea; 3 Faculty of Economics, Shiga University, Hikone, Japan; 4 The Center for Public Service, Portland State University, Portland, OR USA

**Keywords:** Green infrastructure, Flood resilience, Flood risk management, Resilience cost, Spatial heterogeneity

## Abstract

The aim of this study is to empirically assess the effectiveness of green infrastructure (GI) in South Korea as a way to enhance flood resilience. We applied a spatial analytical approach to examine the impact of GI on flood resilience. Specifically, we tested spatial dependence with Moran’s *I* and local indicators of spatial association, addressing it with spatial lag and spatial error models. We compiled government data on GI and other regional attributes for 226 administrative districts in South Korea. Unlike previous studies that focused only on damage costs, this study incorporated both damage and recovery costs to calculate flood resilience costs. The findings show that flood resilience costs and GI distribution exhibit substantial spatial heterogeneity and significant spatial autocorrelation across local governments. Moreover, GI reduces flood resilience costs, although its effectiveness varies by type. No spatial spillover effect of GI on neighboring areas was identified, indicating that the impact of GI is localized and spatially confined. These results provide empirical evidence that GI plays a crucial role in strengthening local resilience against flood risk. The findings highlight the need to strengthen local resilience through spatially efficient expansion, hydrologically optimized design, and strategic allocation of GI, particularly considering flood vulnerability and interregional GI equity. By quantifying resilience costs and employing spatial analysis, this study advances both the theoretical understanding and the practical application of GI in flood-prone regions.

## Introduction

According to the United Nations Office for Disaster Risk Reduction (UNDRR 2020), the number of disasters nearly doubled from 3656 in 1980–1999 to 6681 in 2000–2019, with approximately half of them being flood-related. With floods becoming more intense and frequent due to climate change, flood risk management has become increasingly important (Birchall and Bonnett 2021; Han 2024). Effective management strategies encompass a wide range of measures that reduce vulnerability and exposure to natural hazards, including both reactive responses and proactive precautionary strategies (Dorji et al. 2024; Han and Kim 2023). To effectively address flood risks and climate-related impacts, strategies should consider regional absorptive, adaptive, and restorative capacity, as well as geographical and climatic characteristics, because these factors significantly influence both the type of disaster and the extent of damage (Cutter et al. 2014; Koh et al. 2010).

Floods are a severe natural disaster, and green infrastructure (GI) has been recognized as an effective nature-based solution (NBS) for enhancing flood resilience (Casteli Figueiredo Gallardo and Bond 2025). GI refers to publicly constructed natural spaces designed for multiple purposes, such as improving air and water systems, providing recreational space, and preserving ecosystems and biodiversity (Arteaga-Zambrano et al. 2025; Benedict and McMahon 2002). By promoting infiltration, storage, and delayed runoff during rainfall events, GI reduces flood peaks and runoff volumes, thereby providing a practical spatial planning approach for improving flood resilience (Lennon et al. 2014; Liu et al. 2014).

Some studies have examined the flood resilience benefits of GI by assessing the economic costs of flood damage (Kim and Kim 2017; Lee 2020; Seo et al. 2016; Sohn et al. 2021). However, few empirical studies comprehensively account for inherent spatial autocorrelation in econometrics models, the differential impact of various GI types, and other factors influencing flood resilience (Sohn et al. 2021; Tran et al. 2020; Wang et al. 2024). Furthermore, most research in South Korea has focused on major metropolitan areas and paid little attention to broader regional contexts (Kim and Kim 2017; Lee 2020). To address these research gaps, this study explored the following research questions: Do costs related to flood resilience and the distribution of GI exhibit spatial heterogeneity across local governments? Does GI reduce the resilience costs associated with flood damage at the local level? If so, how do the effects differ by GI type?

To answer these questions, this study empirically examined the impact of GI on flood resilience using micro-level regional data. Given that flood damage varies across regions and that resilience costs often exhibit spatial patterns among neighboring areas (Chun et al. 2017; Sohn et al. 2021), we applied a spatial analytical method to enhance the accuracy of the analysis. Moreover, this study identified the most effective primary and subtypes of GI for flood resilience. The analysis was based on 2017 data from 226 local governments in South Korea. To comprehensively evaluate regional resilience, we measured flood resilience using the resilience assessment framework developed by Vugrin et al. (2010, 2011). In addition, the analysis incorporated a range of control variables—socioeconomic, institutional, natural, and infrastructural factors—that are closely related to flood resilience but have been insufficiently considered in previous research. The findings of this study will help policymakers develop more robust frameworks for evaluating GI planning within flood risk management.

## Literature Review

Flood resilience is a comprehensive management approach that enhances the capacity to absorb, adapt to, and recover from flood risks by emphasizing both proactive and reactive measures (Driessen et al. 2018; Lennon et al. 2014; Raikes et al. 2023). In coastal and low-lying countries, enhancing flood resilience has been increasingly recognized as an important risk management strategy that can be used to address the escalating frequency and severity of floods due to climate change (Driessen et al. 2018; McClymont et al. 2020). In South Korea, flood damage accounted for about 90% of all disaster losses between 2012 and 2021 (MOIS 2022). Effective measures for flood resilience seek to enhance resilience by improving the absorptive, adaptive, and restorative capacities of local communities (Birchall and Bonnett 2021; Yu et al. 2015).^1^ To ensure their effectiveness, it is essential to consider region-specific attributes, including social, economic, political, climatic, and institutional factors (Cutter et al. 2014; Folke et al. 2010; Koh et al. 2010).

Recently, GI has gained increased attention as an NBS for enhancing flood resilience. GI serves as a multifunctional tool for mitigating flood risk and enhancing resilience; it contributes to carbon reduction and flood prevention (Arteaga-Zambrano et al. 2025; Casteli Figueiredo Gallardo and Bond 2025; Hamel and Tan 2022). GI encompasses not only constructed green spaces but also land-use planning and design techniques like permeable pavement, retention basins, green roofs, and green walls (Drescher and Sinasac 2021; Eckart et al. 2017; Song et al. 2022; Venkataramanan et al. 2019). The classification and scope of GI vary across disciplines and scholars (Benedict and McMahon 2002; Han 2024).

A number of studies have investigated the role of GI in promoting flood resilience and employed spatial analysis to improve the accuracy of empirical estimates. These studies have observed spatial heterogeneity and autocorrelation in flood occurrence and damage, using indicators such as flood history and damage cost (Sohn et al. 2021; Tran et al. 2020; Wang et al. 2024). The frequency of flood-related hazards varies with geographic location and climatic characteristics (Al-Amin et al. 2019; Koh et al. 2010; Mittnik et al. 2020). Even within a single country, the extent of damage is shaped by varying levels of economic development, physical infrastructure, and institutional capacity (Friend and Monech 2013; Koh et al. 2010). This highlights the importance of considering geographical characteristics when analyzing the impact of GI on flood resilience. In South Korea, the construction of GI falls under the jurisdiction of local governments (KLIC nd), suggesting that the level of GI provision is likely to vary depending on local policy priorities (Chi and Zhu 2019; Kim and Lee 2025). Based on this context, we propose the following hypothesis. Hypothesis 1: The distribution of flood resilience costs and GI is expected to exhibit significant spatial heterogeneity across local government areas, reflecting uneven exposure and vulnerability to flood risks.

Previous studies have analyzed the effects of GI on enhancing flood resilience, reflecting the growing urgency of developing effective management strategies to address flood risks. Although some studies have adopted engineering-based approaches—employing equipment and monitoring programs to assess GI’s effectiveness (Schubert et al. 2017; Yeo and Jung 2013)—others have employed data-driven approaches using empirical modeling techniques. For example, Kim and Kim (2017) applied a Tobit model to assess the impact of GI on reducing natural disaster damage in Seoul and six other metropolitan areas. Lee (2020) used the normalized difference vegetation index derived from satellite data to conduct a multiple regression analysis, revealing that broader distribution of GI, particularly in circular patterns, significantly reduced flood damage.

Among the various quantitative approaches, studies employing spatial analysis have been particularly effective at demonstrating the flood damage reduction effect of GI. Sohn et al. (2021) conducted a spatial panel model to investigate the effects of GI area, dispersion, and shape on private property damage from flooding in Texas. Their analysis revealed significant spatial autocorrelation in flood-related property damage and found that increased dispersion of GI corresponded with reduced damage. Tran et al. (2020) and Wang et al. (2024) used geographically weighted regression to show that GI reduced the frequency of waterlogging in Hanoi and mitigated flood risk in Beijing. Similarly, Pallathadka et al. (2022) employed a spatial regression model to demonstrate that higher GI density in Portland was associated with lower risk of flooding. Based on these findings, we propose the following hypothesis. Hypothesis 2: A higher proportion of GI is expected to be negatively associated with flood resilience costs, demonstrating its role in flood resilience at the local government level.

Although studies have found that GI is effective at reducing the severity of flood damage, empirical analyses that assess the effects of different GI types on flood resilience from a policy perspective remain limited. Recent studies indicate that different forms of GI exhibit heterogeneous impacts on flood risk mitigation, highlighting the importance of GI composition and classification in systematic urban design and spatial planning (Rodriguez et al. 2021; Zhang et al. 2021). Some studies have attempted to classify GI and assess the differential impacts of each type. For instance, Seo et al. (2016) used spatial regression to investigate the impact of green areas and parks on natural disaster damage in South Korea in 2013. Green area was found to be associated with increased damage costs, while parks were not statistically significant. Zhang et al. (2021), based on a classification of cultivated lands, gardens, grasslands, and woodlands, found that woodlands were the most effective at improving resilience.

The differential effectiveness of GI types can be attributed to variations in design standards, structural components, and the extent to which hydrological functions are embedded within them. Open spaces designed to facilitate infiltration, detention, and temporary storage of stormwater tend to exhibit better flood-mitigation performance. Studies have demonstrated that bioretention cells in parks are more effective at reducing runoff and peak flows (Rodriguez et al. 2021; Wang et al. 2019). Liu et al. (2014) showed that the integrated application of four GI techniques—green area expansion, concave design, and the installation of detention ponds and permeable pavements—reduced runoff and peak discharge during rainfall events. These findings suggest that the benefits of GI depend not only on its presence but also on the degree to which hydrological functions are incorporated into its design.

In South Korea, the *Act on Urban Parks and Green Areas* (AUPGA), the *Enforcement Rules of the AUPGA*, and the *Detailed Guidelines on Types of Urban Parks and Green Areas* categorize government-provided GI into urban parks and green areas, each with distinct design standards based on their intended purpose (Han 2024; KLIC nd; Song et al. 2022). Among green areas, *buffer green areas* are designed to mitigate pollution, noise, and disaster risks. When established for disaster response, they are subject to minimum width and canopy coverage requirements (KLIC nd). *Urban parks* are institutionally mandated to perform disaster prevention functions alongside recreational functions, as regulations permit up to 50% of the total area to be designated for detention facilities (KLIC nd). *Living-zone parks*, a subtype of parks, are intended to promote citizens’ rest, leisure, and quality of life and are regulated by detailed criteria to ensure adequate coverage for the resident population. *Theme parks* serve distinct purposes including cultural heritage preservation and urban agriculture and may also incorporate disaster prevention.^2^ Given the differences in purpose and variation in design standards across GI types in South Korea, it is reasonable to hypothesize that their impact on flood resilience may vary. Based on this institutional and empirical context, we propose the following hypothesis. Hypothesis 3: The impact of different GI types in reducing flood resilience costs will vary significantly, indicating that specific forms of GI contribute differently to flood resilience.

Furthermore, there remains a need to consider broader components of flood resilience beyond GI. To accurately evaluate the effectiveness of GI, it is essential to account for the diverse factors that influence flood resilience. Prior studies have categorized these factors into socioeconomic, institutional, natural, and infrastructural dimensions. Socioeconomic factors include economics, population density or composition, education, and health-related indicators (Cao et al. 2023; Rahman et al. 2021; Tu et al. 2023). Institutional factors refer to government capacity, including budget allocations and disaster response systems (Lee 2019; Lee and Kwon 2017). Precipitation, the main cause of floods, is typically used as a key natural factor (Liu et al. 2020; Tu et al. 2023). Infrastructural factors include road and sewer density as well as land use patterns (de Bruijn 2004; Rahman et al. 2021). High road density and effective drainage facilities enhance resilience by improving transportation accessibility and enabling timely water drainage (Cao et al. 2023; Lee and Kwon 2017; Zhang et al. 2022). The presence of agricultural land can improve flood resilience by reducing impervious surface area (Liu et al. 2020; Tu et al. 2023). Accordingly, this study incorporates a range of socioeconomic, institutional, natural, and infrastructural factors variables as controls to empirically examine the impact of GI on flood resilience.

## Methods

### Flood Resilience Cost Estimation

To quantify flood resilience at the local government level in South Korea, we estimate flood resilience cost using the resilience assessment framework developed by Vugrin et al. (2010, 2011). This framework adopts an equilibrium resilience perspective, emphasizing the minimization of cumulative resources and efforts required to restore a system to its business-as-usual state after a disruptive event (Lee 2019; Lennon et al. 2014; Proag 2014). Within this framework, *resilience cost* captures the shortfall that arises when a system fails to adequately absorb and recover from a disaster event.^3^ Unlike conventional approaches that focus on the economic costs of disaster damage (Kim and Kim 2017; Seo et al. 2016), this framework evaluates resilience cost by considering both systemic impact (SI) and recovery effort (RE).

SI and RE depend on regional absorptive, adaptive, and restorative capacities (Lennon et al. 2014; Vugrin et al. 2011). SI is commonly defined as the impacts of a disruptive event, including structural degradation, functional impairment, and service interruptions (Vugrin et al. 2011).^4^ Conceptually, it reflects a lack of absorptive and adaptive capacity to withstand direct effects, such that larger SI values indicate lower resilience. RE captures the resources and efforts required to restore system performance, reflecting restorative efficiency in the post-disaster phase (Lee 2019; Yu et al. 2015). It may include both short-term recovery costs and proactive investments aimed at enhancing long-term resilience. However, such investments do not necessarily translate into immediate resilience outcomes due to time lags, and may therefore represent additional expenditures from an equilibrium resilience perspective (Drechsler and Hartig 2011; Hocherman et al. 2025).^5^ Accordingly, larger RE values indicate lower resilience, as they represent resources that would not have been required under a more resilient system.

Flood resilience cost is defined as the sum of SI and RE over the disaster event period, as shown in Eq. (1). SI is operationalized as disaster damage cost, representing economic losses attributable to flood events. RE is measured as post-disaster recovery cost incurred during the initial restoration phase. *t*_*0*_ and *t*_*f*_ represent the start and end of the disaster event period.

1 $${Flood}\,{Resilience}\,{Cost}={\int }_{{t}_{0}}^{{t}_{f}}\left[{SI}\left(t\right)\right]{dt}+{\int }_{{t}_{0}}^{{t}_{f}}\left[{RE}\left(t\right)\right]{dt}={\int }_{{t}_{0}}^{{t}_{f}}{Damage}\,{cost}\left(t\right)+{\int }_{{t}_{0}}^{{t}_{f}}{Recovery}\,{cost}\left(t\right)$$

In South Korea, official damage costs aggregate losses across public and private assets within administrative boundaries, including buildings, agricultural facilities, fish farming equipment, and other spatially distributed public infrastructure (MOIS 2018). Recovery costs are calculated based on financial support provided to both public and private sectors, in accordance with the *Regulations on the Burden Sharing Standards for Natural Disaster Relief and Recovery Costs* (KLIC nd). Recovery costs include the direct recovery expenditures for both sectors that are physical and monetizable, such as facility repair and reconstruction. Recovery costs also include indirect recovery expenditures aimed at stabilizing the livelihoods of disaster victims, including tax relief, reductions in or exemptions from public utility charges, and financial support. These expenditures primarily support post-disaster response rather than proactive investments in future disaster prevention, with budgetary control provisions intended to prevent unlimited fiscal support and constrain recovery spending to short-term restoration (KLIC nd). Non-financial damages, such as human casualties, are not fully captured due to the need for additional valuation data (Yu et al. 2015).^6^

To ensure regional comparability and satisfy normality assumptions, the flood resilience cost variable is area-normalized and log-transformed. Accounting for the affected area is essential when assessing disaster-related damage in economic terms, as climate-related impacts are closely linked to the spatial extent and magnitude of physical damage (Arrighi et al. 2018; Merz et al. 2010). Accordingly, we estimate flood resilience costs by dividing the total damage and recovery cost (in 1,000 KRW) by the area of the administrative district (in km²). The final cost estimation is specified as follows:

2 $${Flood}\,{Resilience}\,{Cost}=\frac{{\int }_{{t}_{0}}^{{t}_{f}}{Damage}\,{cost}\left(t\right)+{\int }_{{t}_{0}}^{{t}_{f}}\left[{Direct}\,{recovery}\,{cost}\left(t\right)+{Indirect}\,{recovery}\,{cost}\left(t\right)\right]}{{Area}\,{of}\,{administrative}\,{district}\,(t)}$$

### Model Specification and Data Description

The analysis focused on government-constructed public GI to examine its role in enhancing flood resilience, using regression models with district-level data. To assess variation across GI types, GI is classified into three categories based on the AUPGA: total GI, primary types of GI, and subtypes of GI. Model (1) examines the impact of total public GI (*GI_total*). Model (2) focuses on two primary types of GI: green areas (*GI_area*) and urban parks (*GI_park*). Model (3) investigates five subtypes of GI: buffer green areas (*GI_area_buffer*), scenic green areas (*GI_area_scenic*), connecting green areas (*GI_area_connect*), living-zone parks (*GI_park_living*), and theme parks (*GI_park_theme*).

The model also includes control factors related to flood resilience, categorized into socioeconomic, institutional, natural, and infrastructural dimensions. Socioeconomic factors include population density (*den*) and gross regional domestic product (*grdp*). Institutional factors include the local government’s disaster budget (*budget*) and the number of disaster emergency agencies (*agen*). The precipitation (*rain*), the primary cause of flooding, is included as a natural factor. Infrastructural factors consist of storm sewer density (*sewer*), road density (*road*), and the agricultural land (*agri*). The regression specification is given in Eq. (3).

3 $$\begin{array}{c}{{Flood}\,{Resilience}\,{Cost}}_{i}={\beta }_{0}+{\beta }_{1}{{GI}}_{i}+{\beta }_{2}{{den}}_{i}+{\beta }_{3}{{grdp}}_{i}+{\beta }_{4}{{bud}}_{i}+{\beta }_{5}{{agen}}_{i}+{\beta }_{6}{{rain}}_{i}\\ +{\beta }_{7}{{sewer}}_{i}+{\beta }_{8}{{road}}_{i}+{\beta }_{9}{{agri}}_{i}+{\varepsilon }_{i}\end{array}$$

We compiled data from 226 administrative districts in South Korea for 2017, the most recent year with comprehensive data available. The final sample includes 208 districts, as spatial regression requires listwise deletion of observations with missing data (Boehmke et al. 2015). Detailed information on all variables is provided in Table 1. Given the spatial nature of the data and potential spatial dependence, the next subsection applies spatial econometric techniques to further examine the relationship between GI and flood resilience.

**Table 1:** Variables measurement and descriptive statistics

Variables				Measurement (Unit)	Mean (Std. dev.)	Range	Literature sources	Data sources
Dependent variable								
*cost*			Flood resilience cost	Log-transformed flood-related damage and recovery cost for local governments/Total district area (1,000 KRW/km^2^)	2.139 (4.737)	–2.303 to 12.069	Vugrin et al. (2010, 2011)	MOIS (2018)
Independent variables								
GI	Total	*GI_total*	Sum of public GI	(Area of GI/Total district area)$\times$100 (%)	3.507 (5.239)	0.000–29.437	Han (2024), KLIC (nd), Song et al. (2022)	LX (2017a), KOSTAT (nd)
	Primary	*GI_area*	Green areas	(Green areas/Total district area)$\times$100 (%)	0.494 (0.728)	0.000–6.247		
		*GI_park*	Urban parks	(Urban parks/Total district area)$\times$100 (%)	3.011 (4.961)	0.000–28.971		
	Subtypes	*GI_area_buffer*	Buffer green areas	(Buffer green areas/Total district area)$\times$100 (%)	0.339 (0.518)	0.000–3.482		
		*GI_area_scenic*	Scenic green areas	(Scenic green areas/Total district area)$\times$100 (%)	0.131 (0.369)	0.000–5.030		
		*GI_area_connect*	Connecting green areas	(Connecting green areas/Total district area)$\times$100 (%)	0.024 (0.071)	0.000–0.568		
		*GI_park_living*	Living-zone parks	(Living-zone parks/Total district area)$\times$100 (%)	2.374 (4.129)	0.000–28.124		
		*GI_park_theme*	Theme parks	(Theme parks/Total district area)$\times$100 (%)	0.634 (1.657)	0.000–11.548		
Control variables								
Socioeconomic		*den*	Population density	1000 inhabitants/Total district area (1000 persons/km^2^)	4.028 (6.325)	0.020–27.289	Cao et al. (2023), Rahman et al. (2021), Tu et al. (2023), Lee (2019), Lee and Kwon (2017), Liu et al. (2020)	KOSTAT (nd), Local governments
		*grdp*	GRDP	GRDP (100 billion KRW)	80.535 (104.179)	4.517–680.854		
Institutional		*bud*	Budget for disaster	(Safety and disaster sector budget/Total local government budget)$\times$100 (%)	1.877 (1.606)	0.080–8.890		
		*agen*	Disaster agency	Number of police and fire stations in the district (No.)	17.549 (8.529)	2.000–68.000		
Natural		*rain*	Precipitation	Average annual precipitation in the district (mm)	81.923 (16.803)	45.200–131.300		KMA (2017), KOSTAT (nd)
Infrastructural		*sewer*	Storm sewer density	Total storm sewer length/Total district area (m/km^2^)	1,473.495 (2,714.514)	0.000-22,699.290		LX (2017b, 2017c)
		*road*	Road density	Total road length/Total district area (km/km^2^)	3.789 (12.992)	0.023–191.562		
		*agri*	Agricultural land	(Agricultural land area/Total district area)$\times$100 (%)	16.206 (11.365)	0.000–50.194		

Most socioeconomic and institutional data were obtained from KOSTAT (nd) for metropolitan cities and provinces. When unavailable, data from local governments were used (e.g., Daegu Metropolitan City nd; Seoul Metropolitan City nd)

### Spatial Econometric Analysis

To account for spatial heterogeneity and spatial autocorrelation, we employ both ordinary least squares (OLS) regression and spatial econometric models to assess the effects of GI on flood resilience. Spatial autocorrelation—i.e., correlation among neighboring areas—is a common feature of geographical data (Cliff and Ord 1970; Sohn et al. 2021). Because global models such as OLS assume independence, homoscedasticity, and normality of errors, the presence of spatial autocorrelation can lead to biased estimations (Balaguer-Coll et al. 2019; Kim and Lee 2025). We therefore test for spatial clustering and, where necessary, apply spatial econometric models (Chi and Zhu 2019). To this end, we begin by visualizing spatial distributions using quartile-based mapping and conducting spatial autocorrelation diagnostics to identify spatial heterogeneity, which directly relates to Hypothesis 1.

**Fig. 1:**
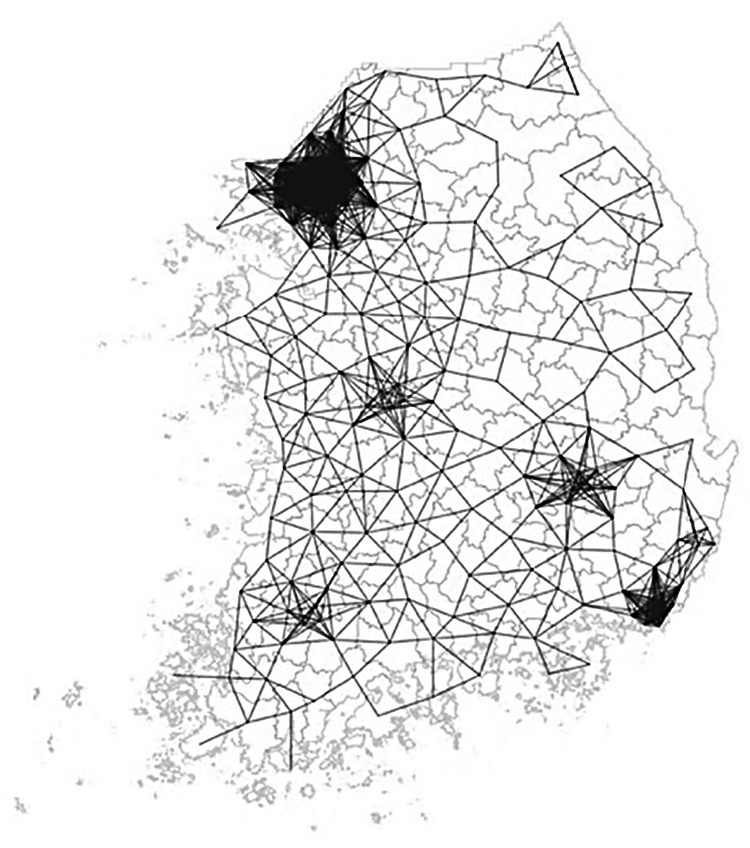
Connectivity map of the distance-band spatial weight matrix

Neighborhood relationships are defined using a distance-band spatial weights matrix, with thresholds ranging from approximately 3 km (3,368.178 m) to 36 km (36,097.2 m), ensuring that all local governments have at least one neighbor based on Euclidean distance (Anselin 2020). Because South Korea includes island regions located far from the mainland, contiguity-based weight matrices can distort neighborhood definitions. A distance-based approach is therefore adopted to better reflect the country’s geographic context, consistent with prior disaster-related spatial analyses (Seo et al. 2016). Figure 1 illustrates the resulting connectivity structure. As a robustness check, alternative k-nearest neighbors (KNN) matrices are also constructed. Spatial autocorrelation was assessed using Moran’s *I* and local indicators of spatial association (LISA), with results reported in Spatial Heterogeneity of Flood Resilience Costs and GI section.^7^

Next, we apply spatial regression models to account for spatial autocorrelation, specifically the spatial lag model (SLM) and the spatial error model (SEM). The SLM is appropriate when spatial dependence appears in the dependent variable, whereas the SEM captures dependence in the error terms (Chi and Zhu 2019). Under the SLM, the effects of GI can be decomposed into direct, indirect, and total effects, enabling identification of spatial spillover (Golgher and Voss 2016; LeSage and Pace 2009). The direct effect captures within-district impacts, the indirect effect reflects spillover to neighboring areas, and the total effect is their sum. Detailed specifications and the derivation are provided in Appendix A.

Model selection is based on the robust Lagrange multiplier (LM) test (Anselin 1988; Chi and Zhu 2019). A significant robust LM_lag_ statistic supports the SLM; otherwise, the SEM is preferred. Because the standard LM test may produce spurious significance under spatially correlated errors, it can falsely indicate lag dependence (Anselin 1988; Anselin et al. 1996). In such cases, both LM_lag_ and LM_error_ may appear significant, requiring the use of the robust LM test. This procedure ensures that estimates of GI effects (Hypotheses 2 and 3) are not biased by unobserved spatial dependence, thereby allowing more accurate identification of both overall and type-specific impacts. This study utilized QGIS 3.28 for data mapping and GeoDa 1.14, along with R packages “spdep” and “spatialreg,” for spatial analysis.

## Results

### Spatial Heterogeneity of Flood Resilience Costs and GI

**Fig. 2:**
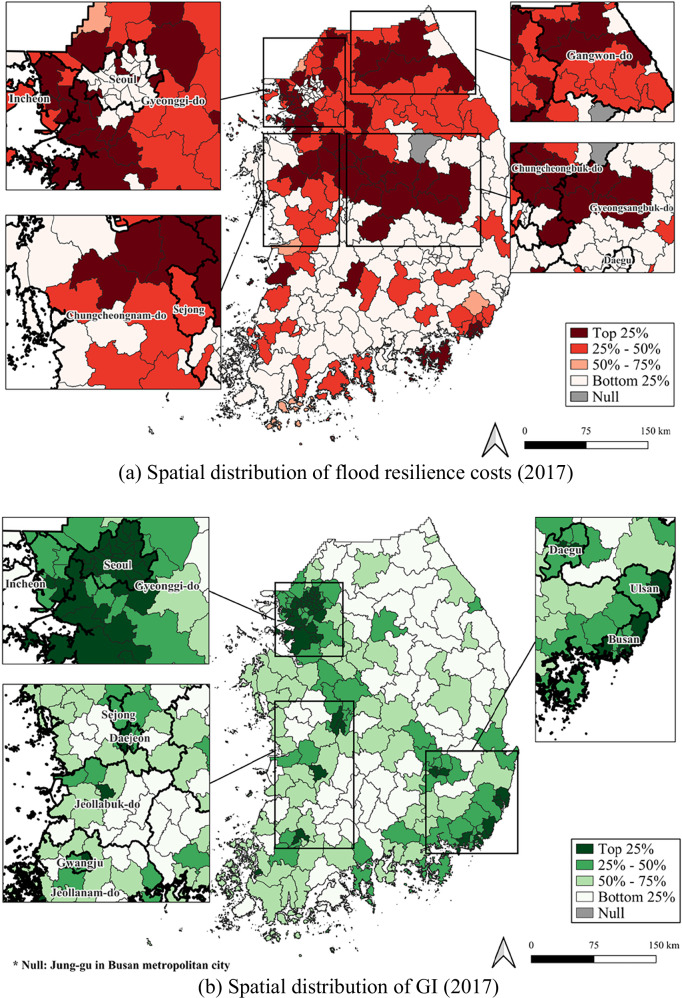
Flood resilience costs and GI distribution

We first mapped flood resilience costs and GI distribution to examine spatial heterogeneity across 226 local governments in South Korea. Figure 2 presents the spatial distribution of flood resilience costs and GI rates using quartile classifications. The results revealed substantial spatial heterogeneity among local governments. High flood resilience costs were concentrated in the northern region, while the proportion of GI was higher in metropolitan areas, including Seoul Metropolitan City, Gyeonggi-do Province, and other metropolitan cities such as Incheon, Daejeon, Gwangju, Daegu, Ulsan, and Busan.^8^

**Fig. 3:**
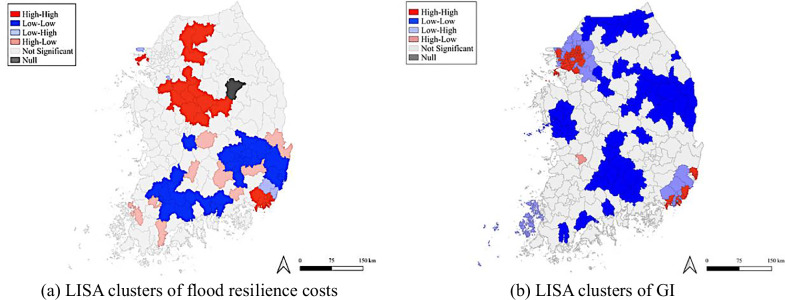
LISA cluster map

Moran’s *I* and LISA analyses were then conducted to evaluate spatial dependence. The Moran’s *I* values for flood resilience costs and GI were 0.356 and 0.312 (*p* < 0.05), respectively, indicating statistically significant positive spatial autocorrelation at the local government level. These results suggest that flood resilience costs and GI in neighboring areas tend to exhibit similar spatial patterns. The LISA results for flood resilience costs further revealed spatial clusters, with HH clusters primarily concentrated in the northern areas and LL clusters in the southern areas (Fig. 3a). For GI, HH clusters were concentrated in metropolitan areas, including Seoul, Gyeonggi-do, Busan, and Ulsan, whereas LL clusters were largely observed in non-metropolitan regions such as Gangwon-do, Chungcheongnam-do, Gyeongsangbuk-do, Gyeongsangnam-do, and Jeollabuk-do (Fig. 3b). This pattern indicates a clear metropolitan–non-metropolitan divide in the spatial distribution of GI, with important implications for GI resource allocation. Overall, these diagnostics provide empirical support for Hypothesis 1, which posited that the distribution of flood resilience costs and GI exhibits significant spatial heterogeneity across local government areas, reflecting uneven exposure and vulnerability to flood risks and climate-related impacts.

### Impact of GI on Flood Resilience Costs

#### Estimation Results

Table 2 presents the estimation results. Results from both OLS and spatial models show consistent statistical significance, indicating that increased GI in a local government is associated with lower flood resilience costs. Based on model fit validation tests (Appendix C), we selected the SLM, which accounts for spatial autocorrelation in the dependent variable, as the preferred specification. The SLM estimates for total GI confirm its effectiveness at reducing flood resilience cost, showing that a 1% increase in total GI is associated with an approximate 16% reduction in costs. This finding aligns with studies that investigated the relationship between GI and flood-related economic losses (Lee 2020; Sohn et al. 2021).

**Table 2:** Regression results

Variables		OLS			SLM			SEM		
		(1)	(2)	(3)	(1)	(2)	(3)	(1)	(2)	(3)
GI	*GI_total*	–0.171^*^ (0.089)	–	–	–0.160^**^ (0.080)	–	–	-0.153^*^ (0.079)	–	–
	*GI_area*	–	0.150 (0.494)	–	–	0.216 (0.440)	–	–	0.188 (0.442)	–
	*GI_area_buffer*	–	–	–0.018 (0.681)	–	–	-0.062 (0.600)	–	–	-0.215 (0.590)
	*GI_area_scenic*	–	–	0.752 (1.043)	–	–	1.090 (0.920)	–	–	1.358 (0.904)
	*GI_area_connect*	–	–	–2.015 (5.028)	–	–	–2.703 (4.432)	–	–	-3.605 (4.415)
	*GI_park*	–	-0.182^**^ (0.091)	–	–	-0.172^**^ (0.081)	–	–	–0.165^**^ (0.080)	–
	*GI_park_living*	–	–	-0.179^*^ (0.102)	–	–	–0.161^*^ (0.090)	–	–	–0.148^*^ (0.088)
	*GI_park_theme*	–	–	–0.189 (0.217)	–	–	–0.211 (0.191)	–	–	-0.226 (0.188)
Socioeconomic	*den*	–0.070 (0.082)	–0.071 (0.082)	–0.058 (0.087)	–0.059 (0.073)	–0.060 (0.073)	–0.040 (0.077)	–0.048 (0.084)	–0.049 (0.084)	–0.021 (0.087)
	*grdp*	0.001 (0.003)	–0.000 (0.004)	–0.001 (0.004)	0.000 (0.003)	–0.001 (0.003)	–0.002 (0.004)	0.001 (0.003)	–0.001 (0.004)	–0.002 (0.004)
Institutional	*bud*	0.048 (0.181)	0.045 (0.181)	0.049 (0.183)	0.070 (0.161)	0.066 (0.161)	0.074 (0.161)	0.059 (0.158)	0.058 (0.158)	0.064 (0.157)
	*agen*	0.055 (0.042)	0.059 (0.042)	0.064 (0.044)	0.056 (0.037)	0.061 (0.038)	0.068^*^ (0.039)	0.038 (0.037)	0.043 (0.037)	0.051 (0.039)
Natural	*rain*	0.133^***^ (0.017)	0.133^***^ (0.017)	0.133^***^ (0.018)	0.102^***^ (0.017)	0.102^***^ (0.017)	0.103^***^ (0.017)	0.124^***^ (0.020)	0.122^***^ (0.021)	0.123^***^ (0.021)
Infrastructural	*sewer*	0.001^***^ (0.000)	0.001^***^ (0.000)	0.001^***^ (0.000)	0.001^***^ (0.000)	0.001^***^ (0.000)	0.001^***^ (0.000)	0.001^***^ (0.000)	0.001^***^ (0.000)	0.001^***^ (0.000)
	*road*	–0.008 (0.023)	–0.010 (0.023)	–0.011 (0.024)	–0.009 (0.021)	–0.012 (0.021)	–0.012 (0.021)	–0.009 (0.020)	–0.011 (0.021)	–0.012 (0.021)
	*agri*	0.038 (0.031)	0.037 (0.031)	0.038 (0.032)	0.035 (0.028)	0.033 (0.028)	0.034 (0.028)	0.063^*^ (0.036)	0.060^*^ (0.036)	0.065^*^ (0.037)
Constant		–10.883^***^ (1.848)	–10.919^***^ (1.851)	–11.047^***^ (1.879)	–9.074^***^ (1.699)	–9.108^***^ (1.693)	–9.262^***^ (1.702)	–10.013^***^ (2.076)	–9.902^***^ (2.077)	–10.141^***^ (2.099)
*ρ* (Rho)		–	–	–	0.458^***^ (0.080)	0.460^***^ (0.080)	0.467^***^ (0.079)	–	–	–
λ (Lambda)		–	–	–	–	–	–	0.517^***^ (0.084)	0.517^***^ (0.084)	0.535^***^ (0.082)
No. of observations		208	208	208	208	208	208	208	208	208

***, **, and * indicate statistical significance at the 1%, 5%, and 10% levels, respectively

Based on the assumption that the effect of GI on flood resilience costs varies by type, we applied type-specific analytical models (i.e., Models (2) and (3)). Among the primary GI types, urban parks significantly reduced flood resilience cost, but green areas did not show a statistically significant effect. A 1% increase in the proportion of urban parks was associated with an estimated 17.2% reduction in flood resilience costs. The result for urban parks differs from previous studies, which found no significant impact (Seo et al. 2016). However, direct comparisons are limited, as this study considers both damage and recovery costs and employs more recent data, perhaps capturing the effects of GI expansion through the addition of urban parks. In Model (3), most GI subtypes were not statistically significant for flood resilience costs once spatial autocorrelation was controlled for. However, living-zone parks within the urban park category remained statistically significant, indicating that a 1% increase in their proportion was associated with an estimated 16.1% reduction in flood resilience costs. This result aligns with previous studies comparing the effectiveness of different GI types at enhancing flood resilience (Rodriguez et al. 2021; Seo et al. 2016; Zhang et al. 2021). These GI effects were robust under sensitivity analyses using alternative disaster exposure–related denominators and KNN weights (Appendix D).

Regarding the control variables, average annual precipitation (natural factor), storm sewer density, (infrastructural factor), and the number of disaster emergency agencies (institutional factor) significantly affected flood resilience costs in Model (3). Average annual precipitation and storm sewer density significantly increased flood resilience costs in both the global and spatial approaches. Precipitation directly contributes to flood events, so regions with more precipitation tend to incur higher flood resilience costs (Omitaomu et al. 2021). Similarly, storm sewers were positively associated with resilience costs, possibly reflecting flood events that exceeded system capacity and post-flood infrastructure repairs (Kim and Kim 2017; Lee and Kwon 2017). Moreover, the number of disaster emergency agencies in Model (3) were positively associated with flood resilience costs (*p* < 0.1). The observed impact of disaster emergency agencies may reflect the concentration of institutional resources in areas with pre-existing geographic vulnerability to flooding, such as regions with prior flood histories, as governed by the *Framework Act on Firefighting Services* and *Regulations on the Establishment of Local Fire Agencies* (KLIC nd). The significance of variables such as disaster emergency agencies in the spatial analysis—despite their insignificance in the OLS analysis—suggests that the SLM provides a better fit than traditional global approaches.

**Fig. 4:**
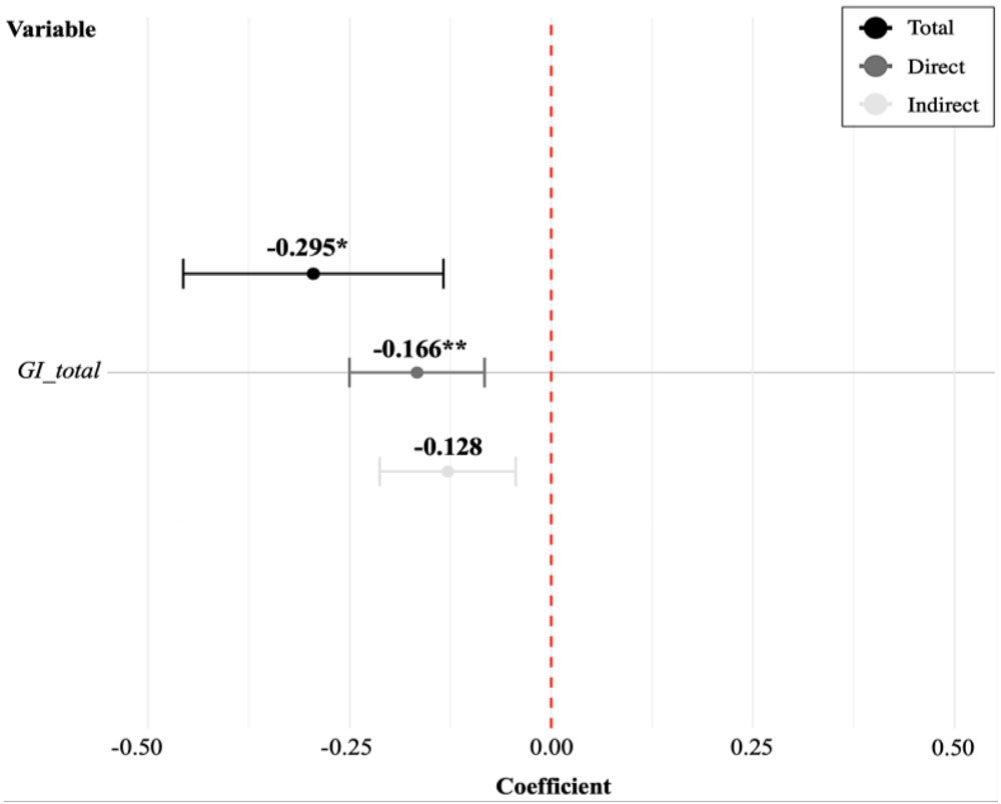
Coefficient plot based on estimated total, direct, and indirect effects of total GI on flood resilience costs (SLM results)

**Fig. 5:**
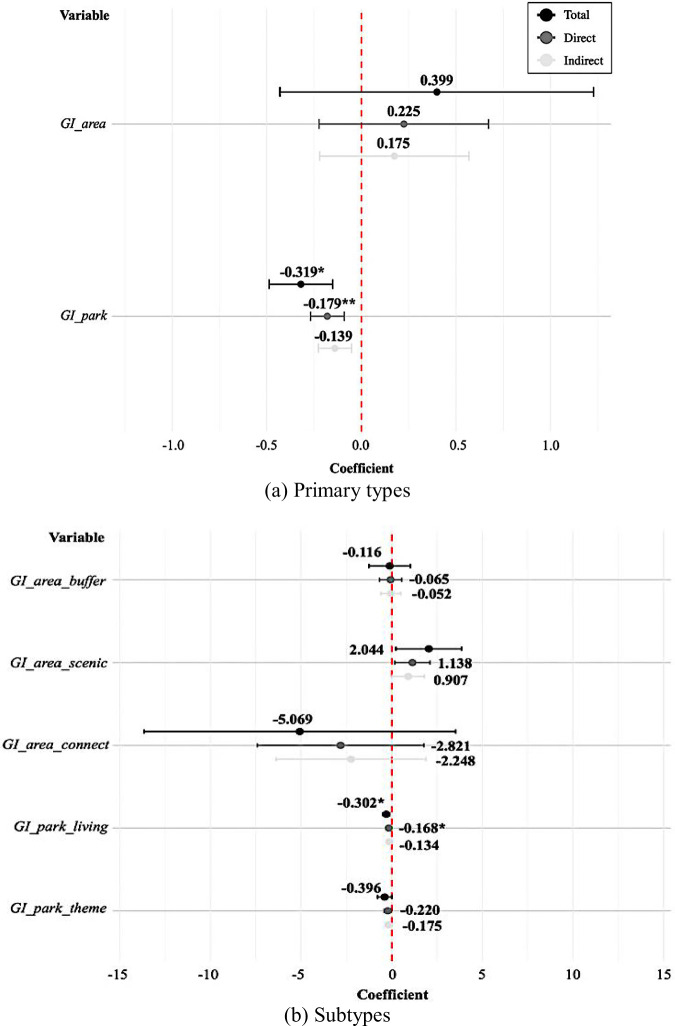
Coefficient plot based on estimated total, direct, and indirect effects of different GI types on flood resilience costs: **a** primary types—green areas and urban parks; **b** subtypes—buffer, scenic, connecting green areas, living-zone, and theme parks

Based on findings from the preceding SLM-based analysis, Figs. 4 and 5 provide an in-depth estimation of how total and specific types of GI affect flood resilience costs, with emphasis on both direct and spillover effects. Figure 4 displays the total, comprehensive effect of GI based on the SLM results. For total GI, the overall effect was a reduction of approximately 29.5% with an increase in GI (*p* < 0.1). The direct effect –0.166 (*p* < 0.05) was observed for total GI, representing approximately 56.3% of the overall effect. This indicates a 16.6% cost reduction with no significant indirect effects. The results suggest that the impact of GI is spatially confined and does not spillover into adjacent regions. Therefore, these findings support Hypothesis 2, which posited that a higher proportion of GI is associated with lower flood resilience costs at the local government level, thereby confirming GI’s role as an effective measure for flood risk management.

Figure 5 presents type-specific effects of GI. As shown in (a), among the primary types of GI, increases in urban park rates are associated with an approximate 31.9% reduction in flood resilience costs (*p* < 0.1). The direct effect was –0.179 (*p* < 0.05), reflecting a cost reduction of approximately 17.9% and accounting for 56.1% of the total effect. Moreover, (b) shows that, among GI subtypes, increased coverage of living-zone parks led to an estimated 30.2% reduction in flood resilience costs (*p* < 0.1). The direct effect of more living-zone parks among GI subtypes was an estimated 16.8% reduction in flood resilience costs (*p* < 0.1), representing approximately 55.6% of the total effect. Although indirect effects contributed substantially to the total, they were not statistically significant. To summarize, increases in the proportion of urban parks, especially living-zone parks, contributed to enhanced flood resilience, whereas other GI types exhibited no statistically significant impact. Moreover, the total and direct effects varied across GI subtypes. Therefore, these findings support Hypothesis 3, indicating that the effectiveness of GI differs greatly across types, with urban parks—particularly living-zone parks—playing a distinct role in localized resilience enhancement.

## Discussion

In formulating flood resilience, it is crucial to consider regional characteristics and develop context-specific policy measures (Birchall and Bonnett 2021; Friend and Monech 2013). GI has been recognized as an effective NBS for flood risk management due to its capacity to mitigate the intensity and damage of floods (Kim and Kim 2017; Lee 2020; Schubert et al. 2017; Sohn et al. 2021). Building on this empirical evidence, this study draws several policy implications.

First, urban planning for flood resilience must consider feasible, context-sensitive strategies for expanding GI. Hypothesis 2 predicted that GI areas within local governments would have a positive effect on flood resilience. The empirical results confirmed that GI plays a significant role in reducing flood resilience costs, implying that public GI provision is an effective measure for improving local capacity to manage flood risks. National trends over the previous period (2013–2017), however, show a decline in GI area in five metropolitan cities and four provinces (Appendix E). In the subsequent period (2017–2021), although some metropolitan cities experienced modest increases in GI area, Daegu, Daejeon, and Ulsan continued to experience declines, alongside a rise to eight provinces showing pronounced GI decreases. This decline is closely related to urban development trends, as rising development permits and conversion of land to residential and industrial uses have coincided with a decline in overall GI (KOSTAT nd). These dynamics further constrain land availability for GI, especially in densely populated regions facing intense development pressure and scarce developable land (Haaland and van Den Bosch 2015). Consequently, securing suitable land for GI expansion is a prerequisite for the successful implementation of GI-based resilience strategies.

In this context, confining GI expansion to publicly owned land may be insufficient, underscoring the need for more effective incentive mechanisms to enable expanded utilization of private land. Since 2009, the central government has implemented the Private-Initiated Park Development Project to promote GI provision by the private sector (Kim 2022). In addition, some local governments provide floor area ratio incentives when privately owned buildings incorporate public green open spaces (Seoul Metropolitan Government 2024). However, in areas where land prices and development pressures are already high, such incentives tend to be leveraged primarily to advance private development interests rather than to promote the provision of functionally effective and space-efficient GI (Kim 2022; Oh and Kim 2025). Future strategies should adopt more differentiated incentive designs that account for local land availability constraints and existing levels of GI provision.

Second, policy strategies should focus on enhancing GI’s flood-mitigation functions rather than solely emphasizing its quantitative expansion. In line with Hypothesis 3, findings from Models (2) and (3) reveal that most GI types do not exhibit statistically significant effects on flood resilience. This may be attributed to the fact that the prevention functions of other GI subtypes, except living-zone parks, are not fully implemented in practice. Under the current institutional framework, living-zone parks are the only type of GI that appear to function as effective measures, as they are more directly equipped with design features related to flood risk mitigation. The enforcement rules and guidelines of South Korea’s AUPGA include standards for the construction of detention facilities in urban parks, as well as minimum size requirements for living-zone parks to ensure sufficient space for such facilities (KLIC nd). These provisions ensure that living-zone parks can accommodate hydrological functions, such as detention and temporary storage, at an efficient spatial scale (Liu et al. 2014; Rodriguez et al. 2021).

As a feasible policy alternative to maximize the flood resilience benefits of GI, it is necessary to further refine and clarify design standards for GI subtypes. This requires the integrated design of hydrological functions alongside consideration of spatial configuration. For example, existing guidelines for buffer green areas for disaster prevention emphasize canopy coverage (KLIC nd), which is more effective for mitigating heat stress and absorbing air pollutants than for flood risk mitigation (Mori et al. 2018). Moreover, most theme parks do not specify minimum size requirements, potentially constraining their capacity to accommodate effective detention facilities, especially in smaller parks. Therefore, design guidelines for buffer green areas and other theme parks should be complemented by explicit standards addressing ground-level permeability and infiltration capacity, including low-impact development techniques and area-based standards for permeable and infiltrative surfaces.

Third, strategic consideration of spatial arrangements—particularly in site selection—is essential. It can be operationalized through spatial allocation of GI based on flood vulnerability. The mapping results support Hypothesis 1 by showing that high flood resilience costs were concentrated in the northern areas, whereas high levels of GI were dispersed across several metropolitan cities. Moreover, the five regions where GI areas declined prior to 2017—Seoul, Incheon, Busan, Jeollanam-do, and Gyeongsangnam-do—were among the highest quartile of flood resilience costs in 2017. These patterns suggest a potential link between declining GI and increased costs in vulnerable regions, reflecting a mismatch between GI resources and flood vulnerability. These findings imply that GI spatial allocation needs to strengthen early-stage planning procedures to better incorporate flood vulnerability into future policy formulation.

In South Korea, a strategy for GI arrangement that incorporates regional vulnerability remains underdeveloped. In contrast, in countries with advanced GI planning, policy formulation incorporates both quantitative targets for GI expansion and explicit evaluations of flood risk and vulnerability at the local or district level. For example, New York City introduced GI pilots in flood-prone areas after vulnerability assessments, Manchester identified priority sites before construction, and London developed a spatial strategy that considers river and tidal flood risks (Greater London Authority 2012; New York City Government 2010; Song et al. 2022). Drawing on these experiences, local governments need to identify the priority areas through vulnerability assessments and analysis of GI demand prior to GI planning.

Fourth, GI allocation also warrants consideration of equity to address interregional disparities. In line with Hypothesis 1, mapping results indicate that GI is concentrated in major metropolitan areas. This pattern is supported by the LISA analysis, which identified statistically significant HH clusters in Seoul, Gyeonggi-do, Busan, and Ulsan. Given that the GI policy process is primarily undertaken by local governments (KLIC nd), GI provision is inherently shaped by local fiscal capacity. Regions with limited fiscal capacity are thus more likely to experience persistent GI deficits. Consistent with the mapping and LISA results of this study, the 2017 fiscal self-reliance index was highest for local governments in Seoul, Gyeonggi-do, Incheon, and Ulsan, whereas most other provinces ranked near the bottom (KOSTAT nd). Investments in GI are also constrained by population decline in non-metropolitan regions in South Korea (MOIS nd), as the scale and location of public infrastructure are commonly determined based on resident population coverage (KLIC nd; Maruani and Amit-Cohen 2007).

These fiscal and demographic disparities between metropolitan and non-metropolitan regions raise concerns about inequities in GI allocation for flood risk management. According to AUPGA, central government subsidies for GI are limited to land acquisition for park development and the installation of basic facilities like roads and plazas (KLIC nd). This institutional arrangement may make it difficult for local governments to sustain GI maintenance and incorporate flood-mitigation functions such as detention facilities. This highlights the need to expand central government subsidies and introduce explicit equity criteria into interregional GI distribution. Specifically, central government subsidy programs for GI development could be designed by aligning with the Special Accounts for Balanced National Development, allowing differentiated financial support based on local fiscal capacity or regional underdevelopment.

Overall, the findings suggest that effective GI-based flood resilience strategies require a coherent policy approach that integrates considerations of the feasibility of GI expansion, functional performance of GI design, and spatial allocation. Under conditions of land scarcity and fiscal disparity, reliance on quantitative expansion alone is insufficient, underscoring the need for differentiated incentive mechanisms that can mobilize private land in ways that reflect local land availability and existing GI provision. At the same time, refining subtype-specific design standards—particularly through the integration of hydrological functions and spatial configuration—can help ensure that GI operates effectively for flood mitigation. More broadly, incorporating flood vulnerability and GI demand into spatial planning, alongside explicit equity criteria and expanded central government support, is essential to achieving GI-based flood resilience.

## Conclusion

To evaluate the effect of GI on flood resilience, this study employed spatial regression models to investigate the impact of GI on flood resilience costs. We calculated the 2017 flood resilience costs for 226 local governments using the framework for resilience assessment proposed by Vugrin et al. (2010, 2011). Furthermore, we provided evidence of spatial heterogeneity and autocorrelations, highlighting the suitability of the spatial approach for verifying the impact of GI.

The empirical findings indicate that flood resilience costs decrease as the extent of GI areas within local governments increases. This result supports Hypothesis 2, confirming that higher GI coverage contributes to lower flood-related costs. We also found that urban parks and living-zone parks significantly reduce these resilience costs. These findings support Hypothesis 3, demonstrating that different GI types contribute unevenly to flood resilience. Even after accounting for spatial characteristics, however, other subtypes of green areas and urban parks had no significant effect on flood resilience. Moreover, no spatial spillover effects were observed from GI. This finding is particularly significant because it validates the direct and indirect effects of each GI type, aspects that have been largely overlooked in previous studies. In addition, the presence of spatial heterogeneity and autocorrelation confirmed in this study supports Hypothesis 1, emphasizing the uneven distribution of flood risk exposure and GI across local governments.

This study demonstrates that enhancing flood resilience through GI requires context-sensitive, feasible strategies for GI expansion under conditions of limited land availability. Given persistent development pressures and declining public GI areas, effective GI expansion increasingly needs to consider the strategic use of private land, supported by incentive mechanisms that go beyond standard approaches. At the same time, the findings highlight that the effectiveness of GI is shaped by its functional design and spatial arrangement rather than by quantitative expansion alone. Urban parks—especially living-zone parks—demonstrate greater flood-mitigation effectiveness due to design standards and minimum size requirements that enable the integration of detention and infiltration functions. Moreover, the spatial analysis reveals a persistent mismatch between flood vulnerability and GI allocation across regions. This result underscores the need to align GI arrangements with flood vulnerability. To address GI equity across regions, future GI-based flood management strategies should strengthen intergovernmental support to reduce interregional disparities in GI provision.

Alongside the academic contributions of this study, we note several limitations. This study has data-related limitations and measurement constraints associated with the use of government-provided data. Temporal changes and certain GI types, such as green roofs and green walls, could not be considered. Missing observations in some jurisdictions reduced the analytical sample, raising concerns about sample selection bias. Flood resilience was measured solely in terms of economic costs. Further research is required to evaluate the effectiveness of a broader range of additional GI types and to consider their durability and the non-financial dimensions of flood damage, such as loss of life. Flood resilience, in particular, is shaped by additional factors such as communities’ experience with flood recovery and governance, which could be considered in future analyses. Furthermore, although this study controlled for various spatial factors, the distribution of GI poses endogeneity concerns. Pre-existing regional conditions—such as a local government’s fiscal capacity, urban development policies, and flood history—may drive both construction of GI and flood resilience, leading to potential reverse causality. Therefore, future studies could benefit from quasi-experimental designs or instrumental variable strategies to rigorously isolate the causal effect of GI while accounting for selection biases.

## Supplementary information

Supplementary information

## Data Availability

All datasets used in this study are publicly available from official government sources referenced in the manuscript, including the Ministry of the Interior and Safety (MOIS), Statistics Korea (KOSTAT), the Korea Meteorological Administration (KMA), and the Korea Land and Geospatial InformatiX Corporation (LX). As these are open-access public data, they can be freely accessed without restriction. Detailed sources are cited in the Model Specification and Data Description subsection of the manuscript.

## Footnotes

1 Vulnerability reflects the difficulty of coping with such climate-related impacts and is inversely related to resilience (Lennon et al. 2014; Proag 2014).

2 Living-zone parks are governed by minimum size requirements—typically ranging from 1,500 m² to 1,000,000 m²—that are intended to secure adequate space, and they are subject to accessibility-based planning standards. Theme parks designated primarily for disaster prevention serve as evacuation and emergency relief hubs, and do not include recreational or ancillary facilities (KLIC nd).

3 It is inversely related to resilience capacity and should therefore be interpreted with caution.

4 Vugrin et al. (2011) illustrated this concept using a hurricane-induced disruption in the petrochemical supply chain, where SI is defined as the aggregated loss of production value over the disruption period, and RE as the additional costs incurred to compensate for these losses.

5 They are more appropriately interpreted as potential capacity for future periods, rather than as direct indicators of resilience outcomes at the time of the disaster.

6 Such losses are typically valued using approaches such as the value of a statistical life.

7 Moran’s *I* ranges from -1 to +1, indicating spatial dispersion or clustering, respectively. LISA identifies four local spatial association types: high-high (HH), low-high (LH), low-low (LL), and high-low (HL) (Chi and Zhu 2019; de Jong et al. 1984).

8 In 2017, the highest quartile of flood resilience costs was observed in selected districts within Seoul, Incheon, Busan, Gyeonggi-do, Gangwon-do, Chungcheongbuk-do, Chungcheongnam-do, Jeollabuk-do, Gyeongsangbuk-do, and Gyeongsangnam-do. High GI rates were concentrated in Seoul; Incheon; Daejeon; Gwangju; Daegu; Ulsan; Busan; and parts of Gyeonggi-do, Jeollabuk-do, and Jeollanam-do (see Appendix B for the full list of districts).
