# Supplementary material for: The Role of Green Infrastructure in Enhancing Flood Resilience: A Spatial Perspective
Source: Environ Manage. 2026 Apr 21;76(5):169. doi: 10.1007/s00267-026-02470-9 (PMC13099833; doi:10.1007/s00267-026-02470-9)
Supplement: Supplementary file 1 — Supplementary information [file 267_2026_2470_MOESM1_ESM.pdf]

The Role of Green Infrastructure in Enhancing Flood Resilience: A Spatial Perspective

Appendices

A. Model specifications of spatial regression models

Eq. (1) presents the spatial lag model (SLM), which includes the influence of neighboring areas on the dependent variable through a spatially lagged term (ρWY). Here, ρ denotes the spatial autoregressive coefficient of Y, W is the spatial weights matrix, and ε is an error term.

$$Y = \rho WY + \beta X + \varepsilon \tag{1}$$

The SLM enables a more detailed estimation of green infrastructure (GI) impacts by distinguishing between direct and indirect effects. The direct effect represents the impact of GI within local government i on its own flood resilience. The average direct effect is computed as the mean of the diagonal elements of the spatial multiplier matrix $(I-\rho W)^{-1}$, as shown in Eq. (2), where k denotes the k-th explanatory variable:

$$Average\ direct\ effect\ of\ X_k = \frac{1}{n}\beta_k \sum_{i=1}^{n}[(I-\rho W)^{-1}]_{ii} \tag{2}$$

The indirect effect, also known as the spatial spillover effect, represents the impact of GI in local government i on flood resilience in neighboring areas j. The average indirect effect is computed as the mean of the off-diagonal elements of the spatial multiplier matrix, as shown in Eq. (3):

$$Average\ indirect\ effect\ of\ X_k = \frac{1}{n}\beta_k \sum_{i=1}^{n}\sum_{j\neq i}[(I-\rho W)^{-1}]_{ij} \tag{3}$$

The spatial error model (SEM) accounts for spatial autocorrelation in omitted variables (μ). In Eq. (4), μ denotes the spatial error term, and λ represents its spatial autoregressive coefficient.

$$Y = \beta X + \mu,\ \mu = \lambda W\mu + \varepsilon \tag{4}$$

B. Districts in the highest quartile of flood resilience cost and GI distributions in 2017

This appendix provides a detailed list of districts corresponding to Figure 2. In 2017, districts classified in the highest quartile (top 25%) of flood resilience cost were identified across 10 metropolitan cities and provinces, as shown in Table B1. These districts include five in Seoul, 11 in Busan, five in Incheon, 13 in Gyeonggi-do, four in Gangwon-do, seven in Chungcheongbuk-do, three in Chungcheongnam-do, five in Gyeongsangbuk-do, two in Gyeongsangnam-do, and two in Jeollabuk-do.

Table B1 Districts in the highest quartile of flood resilience costs

	Districts	Total
Metropolitan city		
Seoul	Gangdong, Guro, Dobong, Seongdong, Jongno	5
Busan	Saha, Sasang, Seo, Jung, Dong, Busanjin, Dongnae, Yeonje, Suyeong, Nam, Yeongdo	11
Incheon	Bupyeong, Namdong, Seo, Dong, Michuhol	5
Province		
Gyeonggi	Goyang, Bucheon, Gwangmyeong, Siheung, Ansan, Gunpo, Uiwang, Suwon, Hwaseong, Namyangju, Pocheon, Anseong, Yeoju	13
Gangwon	Chuncheon, Gangneung, Inje, Hongcheon	4
Chungcheongbuk	Cheongju, Jeungpyeong, Goesan, Boeun, Yeongdong, Eumseong, Jincheon	7
Chungcheongnam	Asan, Cheonan, Yesan	3
Gyeongsangbuk	Mungyeong, Andong, Sangju, Yecheon, Uiseong	5
Gyeongsangnam	Tongyeong, Geoje	2
Jeollabuk	Jangsu, Buan	2

Note: For brevity, the suffixes “-do,” “-si,” “-gu,” and “-gun” are omitted from administrative unit names.

Next, districts in the highest quartile (top 25%) of GI rates in 2017 were identified, as shown in Table B2. These include 23 districts in Seoul, eight in Busan, four in Daegu, two in Incheon, two in Gwangju, three in Daejeon, four in Ulsan, nine in Gyeonggi-do, and one each in Jeollabuk-do and Jeollanam-do. Districts with high levels of GI are concentrated in metropolitan areas, with Seoul accounting for the largest share, followed by Gyeonggi-do and Busan, indicating a strong metropolitan concentration in GI distribution.

Table B2 Districts in the highest quartile of GI rates

	Districts	Total
Metropolitan city		
Seoul	Jongno, Jung, Yongsan, Seongdong, Gwangjin, Dongdaemun, Jungnang, Seongbuk, Gangbuk, Dobong, Nowon, Eunpyeong, Seodaemun, Mapo, Yangcheon, Gangseo, Yeongdeungpo, Dongjak, Seocho, Gangnam, Songpa, Gangdong, Guro	23
Busan	Gangseo, Nam, Dongnae, Busanjin, Seo, Yeongdo, Haeundae, Gijang	8
Daegu	Nam, Dalseo, Seo, Suseong	4
Incheon	Michuhol, Namdong	2
Gwangju	Buk, Seo	2
Daejeon	Seo, Yuseong, Jung	3
Ulsan	Nam, Dong, Buk, Jung	4
Province		
Gyeonggi	Bucheon, Siheung, Ansan, Gunpo, Suwon, Hwaseong, Osan, Gwacheon, Seongnam	9
Jeollabuk	Jeonju	1
Jeollanam	Mokpo	1

Note: For brevity, the suffixes “-do,” “-si,” “-gu,” and “-gun” are omitted from administrative unit names.

C. Model fit and model selection

Model fit validation was conducted to identify the most appropriate specification. The results of all goodness-of-fit tests show that spatial regression models provide a better fit than the ordinary least squares (OLS) model, which does not adequately account for spatial heterogeneity and spatial dependence in flood resilience costs. The condition numbers in each OLS model were below 30, indicating no issue with multicollinearity (Sahinler and Bek 2002). However, these estimations remain subject to bias because the OLS model fails to meet the basic assumptions of normality and homoscedasticity. The Jarque-Bera test indicates that errors do not satisfy the assumption of normality in all models. Additional tests (Breusch-Pagan, Koenker-Bassett, White) reveal heteroscedasticity issues, except the Breusch-Pagan test for Model (2). Moreover, the data exhibit clear spatial heterogeneity and autocorrelation. In this context, OLS is likely to yield biased results, indicating that an alternative model was required.

Table C1 presents the R^2, log-likelihood, Akaike information criterion (AIC), and Schwarz criterion (SC) values for all models. The R^2 values are consistently higher for all spatial models than for OLS, indicating greater explanatory power. The log-likelihood values of the SLMs are -561.361, -560.985, and -560.318, respectively, exceeding those obtained from the corresponding OLS models. The AIC values of the SLMs are 1144.720, 1145.970, and 1150.640, and the SC values are 1181.440, 1186.020, and 1200.700—each lower than those of the OLS specifications. A model is considered more suitable when log-likelihood value is higher, and the AIC and SC values are lower (Setiawan et al. 2019). The likelihood ratio statistic, which indicates whether the spatial regression model improves model fit, is significant at the 1% level.

Table C1 Comparison of model fits between OLS and spatial models

	OLS			SLM			SEM		
Model	(1)	(2)	(3)	(1)	(2)	(3)	(1)	(2)	(3)
R^2	0.300	0.301	0.303	0.413	0.415	0.419	0.411	0.413	0.422
Log-likelihood	-575.760	-575.530	-575.284	-561.361	-560.985	-560.318	-562.839	-562.533	-561.271
AIC	1171.520	1173.060	1178.570	1144.720	1145.970	1150.640	1145.680	1147.070	1150.540
SC	1204.900	1209.770	1225.290	1181.440	1186.020	1200.700	1179.050	1183.780	1197.270
Likelihood ratio	-	-	-	28.798***	29.089***	29.932***	25.841***	25.994***	28.025***

Note: ***, **, and * indicate statistical significance at the 1%, 5%, and 10% levels, respectively.

Among the spatial models, the SLM is selected as the preferred specification, as the robust LM statistics were statistically significant across all models. This indicates inherent spatial autocorrelation in the dependent variable, even after accounting for spatial autocorrelation in the error term (Anselin et al. 1996). As

shown in Table C2, both the LM_{lag} and LM_{error} values are significant at the 1% level. Only the robust LM_{lag} values remain significant across all models, whereas the robust LM_{error} values do not, suggesting that the SLM is the more appropriate specification. Taken together, these results indicate that incorporating spatial dependence is essential for accurately estimating the impacts of GI on flood resilience, thereby strengthening the empirical basis for Hypotheses 2 and 3.

Table C2 Comparison of spatial regression models

	SLM			SEM		
Model	(1)	(2)	(3)	(1)	(2)	(3)
LM_{lag}	36.171***	36.612***	37.516***	-	-	-
Robust-LM_{lag}	9.978***	10.050***	9.387***	-	-	-
LM_{error}	-	-	-	26.388***	26.759***	28.217***
Robust-LM_{error}	-	-	-	0.194	0.197	0.088

Note: ***, **, and * indicate statistical significance at the 1%, 5%, and 10% levels, respectively.

D. Sensitivity analyses

To verify the robustness of the estimated results in Estimation Results section, we conduct sensitivity analyses using alternative denominators based on exposure-related factors, including population, public infrastructure, and economic exposure. Population exposure is proxied by population density (1,000 persons/km^2), infrastructure exposure by the average area (m^2) per public infrastructure facility, and economic exposure by GRDP per capita (million KRW). This normalization approach follows Vugrin et al. (2011), who emphasized the need to adjust resilience costs when comparing systems of different scales and magnitudes. Previous studies have used population size or economic level as primary exposure-related factors (Lee 2019; Lee and Kwon 2017; Yu et al. 2015); we additionally incorporate the scale of exposed public infrastructure, given that monetized damage and recovery costs in South Korea mostly reflect losses to physical infrastructure rather than human casualties (MOIS 2018; Yu et al. 2015). This includes transportation facilities, electricity, water supply, sewage, and gas systems, as well as schools, public administrative buildings, cultural and sports facilities, and basic environmental facilities as defined by South Korea's *National Land Planning and Utilization Act*.

SLMs results indicate that GI variables remain statistically significant when flood resilience costs are normalized by population density ($p < 0.1$), public infrastructure stock ($p < 0.05$), and economic level ($p < 0.05$), as shown in Table D1. Among GI types, urban parks show consistently significant effects, and within this category, living-zone parks remain significant across most specifications except the population density–based model. These findings are consistent with the main results, suggesting that the estimated effects of GI on flood resilience are robust across alternative normalization strategies.

Table D1 SLM estimates with alternative exposure-based normalizations

	Population			Infrastructure			Economic		
	(1)	(2)	(3)	(1)	(2)	(3)	(1)	(2)	(3)
GI_total	-0.232*	-	-	-0.092**	-	-	-0.209**	-	-
	(0.139)			(0.046)			(0.099)		
GI_area	-	0.063	-	-	0.214	-	-	0.181	-
		(0.768)			(0.252)			(0.548)	
GI_area_buffer	-	-	-0.630	-	-	0.084	-	-	-0.220
			(1.047)			(0.344)			(0.747)
GI_area_scenic	-	-	1.537	-	-	0.685	-	-	1.396
			(1.602)			(0.526)			(1.143)
GI_area_connect	-	-	-1.746	-	-	-1.865	-	-	-3.503
			(7.729)			(2.537)			(5.512)
GI_park	-	-0.241*	-	-	-0.102**	-	-	-0.221**	-
		(0.141)			(0.046)			(0.101)	
GI_park_living	-	-	-0.238	-	-	-0.086*	-	-	-0.213*
			(0.157)			(0.052)			(0.112)
GI_park_theme	-	-	-0.238	-	-	-0.166	-	-	-0.247
			(0.333)			(0.109)			(0.238)
Control variables	Yes	Yes	Yes	Yes	Yes	Yes	Yes	Yes	Yes
ρ (Rho)	0.416***	0.417***	0.422***	0.530***	0.537***	0.542***	0.428***	0.431***	0.436***
No. of observations	208	208	208	208	208	208	208	208	208

Note: ***, **, and * indicate statistical significance at the 1%, 5%, and 10% levels, respectively.

We further employ k-nearest neighbors (KNN) spatial weights matrices to assess the robustness of the estimated spatial spillover effects of GI. Unlike distance-band weights, KNN weights emphasize localized spatial interactions by restricting spatial dependence to a fixed number of geographically closest neighboring jurisdictions. This allows us to assess whether the absence of spillover effects is sensitive to the specification of spatial proximity. Two KNN specifications are considered. First, to capture localized spatial interactions, the number of neighbors is set to k = 3–5, reflecting the modal distribution observed in the distance-band matrix, where the largest share of local governments have five neighbors. Second, a broader specification with k = 13–15 is applied, corresponding to the average number of neighbors (approximately 14) under the distance-band specification.

Under localized KNN specifications (k = 3–5), as shown in Table D2, Moran's I values are 0.482, 0.474, and 0.469, respectively, and statistically significant at the 5% level. Robust Lagrange multiplier (LM) tests consistently indicate that the SLM is the most appropriate model. However, estimation results show that GI variables remain statistically insignificant, and spatial spillover effects are not statistically significant across all models.

Table D2 SLM results with KNN-based spatial weights (k = 3–5)

	k = 3			k = 4			k = 5		
	(1)	(2)	(3)	(1)	(2)	(3)	(1)	(2)	(3)
GI_total	-0.087<br>(0.080)	-	-	-0.097<br>(0.080)	-	-	-0.098<br>(0.080)	-	-
GI_area	-	0.100<br>(0.443)	-	-	0.140<br>(0.442)	-	-	0.102<br>(0.442)	-
GI_area_buffer	-	-	-0.163<br>(0.603)	-	-	-0.088<br>(0.602)	-	-	-0.134<br>(0.603)
GI_area_scenic	-	-	1.042<br>(0.923)	-	-	1.032<br>(0.923)	-	-	0.937<br>(0.925)
GI_area_connect	-	-	-3.287<br>(4.444)	-	-	-3.336<br>(4.444)	-	-	-2.938<br>(4.453)
GI_park	-	-0.094<br>(0.081)	-	-	-0.105<br>(0.081)	-	-	-0.105<br>(0.081)	-
GI_park_living	-	-	-0.087<br>(0.090)	-	-	-0.098<br>(0.090)	-	-	-0.097<br>(0.091)
GI_park_theme	-	-	-0.104<br>(0.192)	-	-	-0.118<br>(0.192)	-	-	-0.126<br>(0.193)
Control variables	Yes	Yes	Yes	Yes	Yes	Yes	Yes	Yes	Yes
ρ (Rho)	0.383^{***}	0.381^{***}	0.388^{***}	0.414^{***}	0.412^{***}	0.419^{***}	0.430^{***}	0.428^{***}	0.433^{***}
No. of observations	208	208	208	208	208	208	208	208	208

Note: ***, **, and * indicate statistical significance at the 1%, 5%, and 10% levels, respectively.

Under the broader KNN specifications (k = 13–15), as shown in Table D3, Moran's I values are 0.381, 0.365, and 0.345, respectively, and statistically significant at the 5% level. Consistent with the previous results, the SLM is selected based on the robust LM test. Urban park variables remain statistically significant under these specifications ($p < 0.1$), with coefficient signs remaining consistent with those observed in the main results. However, spatial spillover effects of GI are not statistically significant across all models. Overall, these findings suggest that the absence of statistically significant GI spillover effects is robust across alternative specifications of spatial weights and is not driven by the choice of a larger distance-band threshold.

Table D3 SLM results with KNN-based spatial weights (k = 13–15)

	k = 13			k = 14			k = 15		
	(1)	(2)	(3)	(1)	(2)	(3)	(1)	(2)	(3)
GI_total	-0.131<br>(0.080)	-	-	-0.134*<br>(0.081)	-	-	-0.138*<br>(0.082)	-	-
GI_area	-	0. 090<br>(0.444)	-	-	0.111<br>(0.448)	-	-	0.087<br>(0.450)	-
GI_area_buffer	-	-	-0.127<br>(0.606)	-	-	-0.119<br>(0.611)	-	-	-0.133<br>(0.615)
GI_area_scenic	-	-	1.028<br>(0.928)	-	-	1.072<br>(0.936)	-	-	1.032<br>(0.941)
GI_area_connect	-	-	-3.885<br>(4.473)	-	-	-3.806<br>(4.512)	-	-	-3.845<br>(4.539)
GI_park	-	-0.138*<br>(0.082)	-	-	-0.143*<br>(0.082)	-	-	-0.146*<br>(0.083)	-
GI_park_living	-	-	-0.131<br>(0.091)	-	-	-0.137<br>(0.092)	-	-	-0.141<br>(0.092)
GI_park_theme	-	-	-0.158<br>(0.193)	-	-	-0.154<br>(0.195)	-	-	-0.156<br>(0.196)
Control variables	Yes	Yes	Yes	Yes	Yes	Yes	Yes	Yes	Yes
ρ (Rho)	0.550***	0.549***	0.559***	0.543***	0.542***	0.553***	0.531***	0.529***	0.540***
No. of observations	208	208	208	208	208	208	208	208	208

Note: ***, **, and * indicate statistical significance at the 1%, 5%, and 10% levels, respectively.

The choice of spatial weights involves a trade-off between bias and variance. When k is small, KNN-based weights tend to increase variance and sensitivity to local noise, but larger values of k may introduce bias by excessively smoothing spatial variation and averaging out heterogeneous spatial characteristics (Jung et al. 2013). Accordingly, selecting an appropriate k is critical, and previous studies have used model fit criteria such as the AIC and log-likelihood values (Kubara and Kopczewska 2024). Comparisons across specifications indicate that the baseline SLMs using distance-band weights consistently exhibit superior model fit relative to KNN-based models (Table D4). This suggests that distance-band weights are more appropriate for capturing spatial dependence in this study.

Table D4 Model fit of the SLMs with KNN-based spatial weights

	k = 3			*k* = 4			*k* = 5		
Model	(1)	(2)	(3)	(1)	(2)	(3)	(1)	(2)	(3)
Log-likelihood	-561.752	-561.659	-560.944	-561.562	-561.413	-560.750	-561.380	-561.274	-560.690
AIC	1147.500	1149.300	1153.900	1147.100	1148.800	1153.500	1146.800	1148.500	1153.400
	k = 13			*k* = 14			*k* = 15		
Model	(1)	(2)	(3)	(1)	(2)	(3)	(1)	(2)	(3)
Log-likelihood	-561.625	-561.496	-560.738	-563.194	-563.038	-562.287	-564.093	-563.964	-563.240
AIC	1147.200	1149.000	1153.500	1150.400	1152.100	1156.600	1152.200	1153.900	1158.500

This result is also consistent with South Korea’s administrative structure. Metropolitan areas such as Seoul consist of highly fragmented administrative units, with multiple districts densely clustered within a relatively small geographic area. In contrast, non-metropolitan regions consist of much larger administrative units. Under this heterogeneous spatial structure, KNN specifications—which impose a fixed number of neighbors—may oversimplify spatial relationships and artificially equalizing spatial dependence. By contrast, distance-band weights allow the number of neighbors to vary across regions, better reflecting the underlying geographic and administrative configuration.

E. Public GI trends in South Korea (2013–2021)

This appendix presents trends in public GI from 2013 to 2021, measured as the total area (m²) of green areas and urban parks, consistent with the scope of this study. Table E1 indicates that during the pre-2017 period (2013–2017), GI area declined in five metropolitan cities and four provinces, reflecting a widespread contraction of public GI prior to 2017. In the post-2017 period (2017–2021), although several metropolitan cities—such as Seoul, Busan, Incheon, Gwangju, and Sejong—exhibited increases in GI area, metropolitan cities including Daegu, Daejeon, and Ulsan continued to experience persistent or accelerated GI reductions.

At the provincial level, the contraction became more pronounced after 2017, with eight provinces showing net decreases in GI area. Overall, these patterns reveal increasing divergence between metropolitan and non-metropolitan regions and underscore that post-2017 GI expansion has been spatially uneven, with declines concentrated in specific cities and a majority of provinces. From a long-term perspective (2013–2021), all provinces except Gyeonggi-do show an overall decline in public GI area.

Table E1 Trends in public GI areas from 2013 to 2021

	2013	2017	2021	Before 2017 (2013–2017)	After 2017 (2017–2021)	Overall (2013–2021)
	GI area (m^2)			Change direction		
Metropolitan city						
Seoul	55,479,003	53,301,328	55,440,732	−	+	−
Busan	61,788,898	59,686,117	66,363,451	−	+	+
Daegu	33,457,000	32,220,586	24,542,429	−	−	−
Incheon	53,441,771	52,112,917	56,722,798	−	+	+
Gwangju	24,354,482	24,655,168	31,449,634	+	+	+
Daejeon	30,701,067	29,220,899	22,551,632	−	−	−
Ulsan	45,646,641	46,543,507	34,649,466	+	−	−
Sejong	23,111,029	24,389,774	26,857,152	+	+	+
Province						
Gyeonggi	194,090,786	250,776,113	228,609,351	+	−	+
Gangwon	41,523,747	42,417,989	28,925,590	+	−	−
Chungcheongbuk	43,853,819	45,910,807	41,138,657	+	−	−
Chungcheongnam	42,438,746	45,768,102	37,263,234	+	−	−
Jeollabuk	53,050,486	52,103,161	44,007,718	−	−	−
Jeollanam	78,834,812	72,937,671	65,616,002	−	−	−
Gyeongsangbuk	83,060,299	85,318,781	55,925,649	+	−	−
Gyeongsangnam	109,899,535	103,637,519	75,704,527	−	−	−
Jeju	12,089,151	10,552,481	10,780,832	−	+	−

Notes: This table was reconstructed based on green areas and urban parks statistics from KOSTAT (nd). For brevity, the suffix "-do" is omitted from administrative unit names.
